# Reporting of side-effects in clinical trials of psilocybin-assisted psychotherapy for psychiatric conditions: systematic review

**DOI:** 10.1192/bjo.2025.10847

**Published:** 2025-11-03

**Authors:** Jonathon Marinis, Sarah T. Clarke, Alexandre A. Guerin, Adam J. Guastella, Gillinder Bedi

**Affiliations:** Centre for Youth Mental Health, University of Melbourne and Orygen, Australia; Clinic for Autism and Neurodevelopmental Research, Brain and Mind Centre, Children’s Hospital Westmead Clinical School, Faculty of Medicine and Health, University of Sydney, Australia

**Keywords:** Psilocybin-assisted psychotherapy, adverse events, side-effects, quality of reporting, health policy

## Abstract

**Background:**

Psilocybin-assisted psychotherapy (PAP) has gained attention as a promising intervention for conditions including depression, anxiety and post-traumatic stress disorder, but understanding of its side-effects is limited. This review evaluates the quality of side-effects reporting in PAP trials, to guide treatment, policy and research.

**Aims:**

To assess side-effects reporting quality in PAP trials for psychiatric conditions, comparing published articles and ClinicalTrials.gov records.

**Method:**

A PROSPERO-registered review (no. CRD42023458960) included English-language PAP trials (2005–2024) identified via Embase, CENTRAL, PubMed and reference searches. Reporting quality was assessed using the CONSORT Harms extension, categorised as either high (17–21), moderate (12–16), low (7–11) or very low (0–6). Randomised controlled trials underwent risk of bias analysis, and descriptive statistics compared side-effects across sources.

**Results:**

Twenty-four trials were included. Reporting quality was high in six studies, moderate in four, low in nine and very low in five. All randomised controlled trials (*n* = 9) showed high risk of bias for side-effects outcomes. Variability in reporting hindered comparisons between articles and ClinicalTrials.gov, underscoring the need for standardisation. Overall, there was no evidence of systematic underreporting of side-effects in published articles compared with trial registers.

**Conclusions:**

Side-effects reporting in PAP trials is inconsistent but is improving over time. Existing evidence has a high risk of bias. Future trials should align with best-practice guidelines for side-effects reporting. Discussions with patients should prioritise findings from high-quality studies and emphasise the current uncertainty regarding PAP side-effects.

Over the past two decades, clinical research investigating the therapeutic potential of psilocybin as an adjunct to psychotherapy for psychiatric indications has increased markedly.^1^ In parallel, various jurisdictions are moving towards legislating some degree of public access to psilocybin-assisted care. In 2023, the Australian Therapeutic Goods Administration implemented bifurcated scheduling for psilocybin to facilitate its clinical use in treatment-resistant depression by individually authorised psychiatrists.^2^ In the USA, several state-level initiatives enabling legal access to psilocybin have been enacted or are under consideration, with psilocybin services already available in Oregon.^3^ Physicians – including psychiatrists and primary care providers – across a range of jurisdictions will therefore be increasingly required to advise patients who are considering accessing psilocybin for therapeutic purposes.

Because Phase 3 trials of psilocybin-assisted psychotherapy (PAP) – currently under way – have yet to be published, implementation decisions have been made on the basis of evidence from Phase 2 randomised controlled trails (RCTs) and pilot open-label studies.^4–10^ Such studies have suggested that PAP is relatively safe and well tolerated, with promising efficacy signals for treatment-resistant and major depression,^11,12^ as well as for other psychiatric indications including tobacco addiction and body dysmorphic disorder.^7,10^

While several reviews have focused primarily on efficacy,^13–15^ a clear understanding of the potential side-effects of PAP is needed to inform implementation in different populations and contexts, as well as individual treatment decisions.^16^ Six systematic reviews have examined the side-effects of PAP,^17–22^ reporting that common adverse events – including nausea, headache and anxiety – are largely transient,^17,19,20,22^ with little evidence of symptom exacerbation as a function of PAP.^21^ Rare serious adverse events have also been documented, including increased suicidality in patients with depression – a key indication for PAP.^18^

Despite prior reviews noting a need to improve the measurement and reporting of side-effects in studies of PAP,^18^ no previous study has formally assessed the quality of side-effects reporting in this evidence base. Understanding the quality of reporting in this context is important, because low-quality reporting can lead to misinterpretation of research results, ultimately contributing to poor patient care.^23^ Transparent and accurate reporting of methods for characterisation of harms is critical to establish the risk:benefit ratio of new interventions, yet side-effects are often secondary outcomes and receive less focus compared with benefits.^24^ Evaluating the quality of side-effects reporting is particularly important for novel interventions such as PAP, where public and clinician trust hinges on a clear characterisation of both risks and benefits. As demonstrated in other fields – such as vaccine development – assessing and improving the quality of side-effects reporting can enhance transparency, facilitate informed decision-making and ultimately support safe implementation of novel therapies.^24^

We recently assessed the quality of side-effects reporting in research on 3,4-methylenedioxymethamphetamine (MDMA)-assisted psychotherapy (MDMA-AP) for psychiatric indications.^25^ We found that reporting on side-effects of MDMA-AP was largely of low quality, contributing to uncertainty around the risk:benefit profile of this approach. These findings, in addition to similar reports on the side-effects of esketamine^26^ and selective serotonin reuptake inhibitors (SSRIs),^27^ highlight the need to characterise (and improve) the quality of side-effects reporting across the broader psychiatric intervention literature.

Here, we aimed to assess the quality of side-effects reporting in the PAP literature, with ‘side-effects’ used as an umbrella term for outcomes explicitly identified as adverse events in clinical trials of the efficacy, safety and/or tolerability of PAP for psychiatric indications.

## Method

This review is reported in accordance with the PRISMA guidelines.^28^ It was preregistered on PROSPERO (no. CRD42023458960).

### Eligibility criteria

Eligible studies were clinical trials focused on PAP for the treatment of a psychiatric condition, published in an English-language peer-reviewed journal. Exclusion criteria included: (a) non-human studies; (b) human laboratory/Phase 1 studies in healthy participants; (c) studies of PAP for non-psychiatric indications; (d) systematic/meta-analytic reviews; (e) book chapters; (f) commentaries; (g) conference proceedings or abstracts only; (h) reports that did not contain original data; and (i) reports published before 2006, which were excluded to ensure that the review captured only studies using modern clinical trial methodologies and reporting practices.

### Search strategy

The original search was conducted on 9 January 2024 using EMBASE, PubMed and the Cochrane Central Register of Controlled Trials (CENTRAL), using the function presented in Supplementary Table 1 available at https://doi.org/10.1192/bjo.2025.10847. A secondary archival search was conducted on 7 February 2024 of reference lists of articles identified in the initial search, to ensure that no study was missed. A final search was completed on 3 February 2025. The primary reviewer (J.M.) screened all titles and abstracts; this process was repeated by a second, independent reviewer (A.A.G.). Full-text articles were reviewed by two independent reviewers (J.M. and S.T.C.), with any disagreements resolved by either consensus or a third reviewer (G.B.). Articles were screened and full texts stored using Covidence systematic review software (Veritas Health Innovation, Melbourne, Australia; see www.covidence.org (accessed 14 Dec 2023)).

### Data extraction

Information on data extraction is available on page one in the supplementary material. Data were first extracted on 26 March 2024 by primary review (J.M.), and subsequently double-extracted by an independent reviewer (A.A.G.) on 10 March 2025; they were then checked for accuracy and completeness by a third reviewer (S.T.C.).

Consistent with previous studies,^26^ we included non-randomised trials in the quality of side-effects reporting assessment, with results reported separately for RCTs and non-randomised trials.

### Quality of side-effects reporting

In 2004, the Consolidated Standards of Reporting Trials (CONSORT) statement was adapted for safety reporting with the CONSORT Harms extension.^29^ The current systematic review assessed publications between 2006 and the present against these guidelines. The quality of side-effects reporting in all included studies was independently assessed by two reviewers (J.M. and S.T.C.) using the CONSORT Harms 2004 guideline, a 21-item checklist for reporting of side-effects in randomised trials (see Table 1).^29^ We chose to use the 2004 checklist over the recently published edition^30^ because few existing trials were published following publication of the updated checklist in 2023. Cohen’s kappa was calculated to determine reviewer agreement, with a score of 0.80 or greater deemed adequate. Disagreements were resolved through either discussion or mediation by a third reviewer (G.B.). Each checklist item was scored individually (1, adequately reported; 0, inadequately or not reported at all). The total score was calculated by summing all individual scores into a total harms reporting score (THRS). The THRS was then categorised as either very low quality (0–6), low quality (7–11), moderate quality (12–16) or high quality (17–21).^31^

**Table 1:** Quality of reporting criteria CONSORT extension for harm compliance

Section	CONSORT harm recommendations	Detailed items	Compliance of trials (%)
**Title and Abstract**	If the study collected data on harms and benefits, the title or abstract should state so.	1. Adverse events mentioned in title or Abstract	15/24 (63)
**Introduction**	If the trial addresses both harms and benefits, the introduction should state so.	2. Information on adverse events mentioned in the Introduction	4/24 (17)
**Method**	Include a list of adverse events with definitions for each (with attention, when relevant, to grading, expected versus unexpected events, references to standardised and validated definitions and description of new definitions).	3a. Definitions of adverse events mentioned	10/24 (42)
		3b. Whether article mentioned all or selected sample of adverse events	23/24 (96)
		3c. Whether article mentioned the use of a validated instrument to report adverse events severity	9/24 (38)
	Clarify how harms-related information was collected (mode of data collection, timing, attribution methods, intensity of ascertainment and harms-related monitoring and stopping rules, if pertinent).	4a. Description of the mode of data collection (e.g. diaries, phone interviews, face-to-face interviews)	16/24 (67)
		4b. Statement about the timing of collection of adverse events data	19/24 (79)
		4c. Description of how adverse events were attributed to trial drugs	10/24 (42)
		4d. Description of the monitoring plan for harms, and rules for stopping the trial because of harms	9/24 (38)
	Describe plans for presenting and analysing information on harms (including coding, handling of recurrent events, specification of timing issues, handling of continuous measures and any statistical analyses).	5a. Description of the methods for presenting and/or analysing adverse events	10/24 (42)
		5b. Description of approach for the handling of recurrent adverse events	3/24 (13)
**Results**	Describe for each arm the participant withdrawals that are due to harm and the experience with the allocated treatment.	6a. Reported withdrawals because of adverse events in each arm	18/24 (75)
		6b. Reported deaths and serious adverse events	19/24 (79)
	Provide denominators for describing harms.	7a. Provision of denominators for adverse events	11/24 (46)
		7b. Provision of definitions used for analysis set (intention to treat, per protocol, safety data available, unclear)	13/24 (54)
	Present the absolute risk of each adverse event (specifying type, grade and seriousness per arm), and present appropriate metrics for recurrent events, continuous variables and scale variables, whenever pertinent.	8a. Reporting of results separately for each treatment arm	20/24 (83)
		8b. Severity and grading of adverse events	16/24 (67)
		8c. Provision of both number of adverse events and number of patients with adverse events	7/24 (29)
	Describe any subgroup analysis and exploratory analysis for harms.	9. Description of subgroup analysis and exploratory analysis for harms	24/24 (100)
**Discussion**	Provide a balanced discussion of benefits and harms with emphasis on study limitations, generalisability and other sources of information non-harms.	10a. Provision of a balanced view that puts benefits and harms into perspective	10/24 (42)
		10b. Inclusion of limitations of study with respect to harms (e.g. lack of power, short duration of exposure, inconclusive findings, post hoc analysis, generalisability of adverse events data as dependent on clinical setting)	6/24 (25)

CONSORT, Consolidated Standards of Reporting Trials.

### Risk of bias assessment

For the risk of bias assessment, the outcome was side-effects (i.e. not the trial primary outcome). For the nine RCTs, two reviewers (J.M. and S.T.C.) independently assessed risk of bias using the Cochrane Risk of Bias Tool for randomised trials (RoB 2).^32^ Cohen’s kappa was calculated for overall risk of bias to assess reviewer agreement, with a score of 0.80 or greater deemed adequate. Any differences between ratings were resolved through discussion to reach consensus, or mediation by a third reviewer (G.B.).

### Comparison of adverse events in publications and on ClinicalTrials.gov

The US Food and Drug Administration Amendments Act (2007) mandated the reporting of all clinical trial results, including adverse events, in the ClinicalTrials.gov Register (CTR) database.^33^ To assess whether the adverse events reported in published articles align with those recorded in CTR, we compared the total number of serious and ‘non-serious’ adverse events from each of these sources for each trial when available. Previous reviews have suggested underreporting of side-effects in published trials of psychiatric interventions compared with CTR.^26^

## Results

### Study selection

**Fig. 1:**
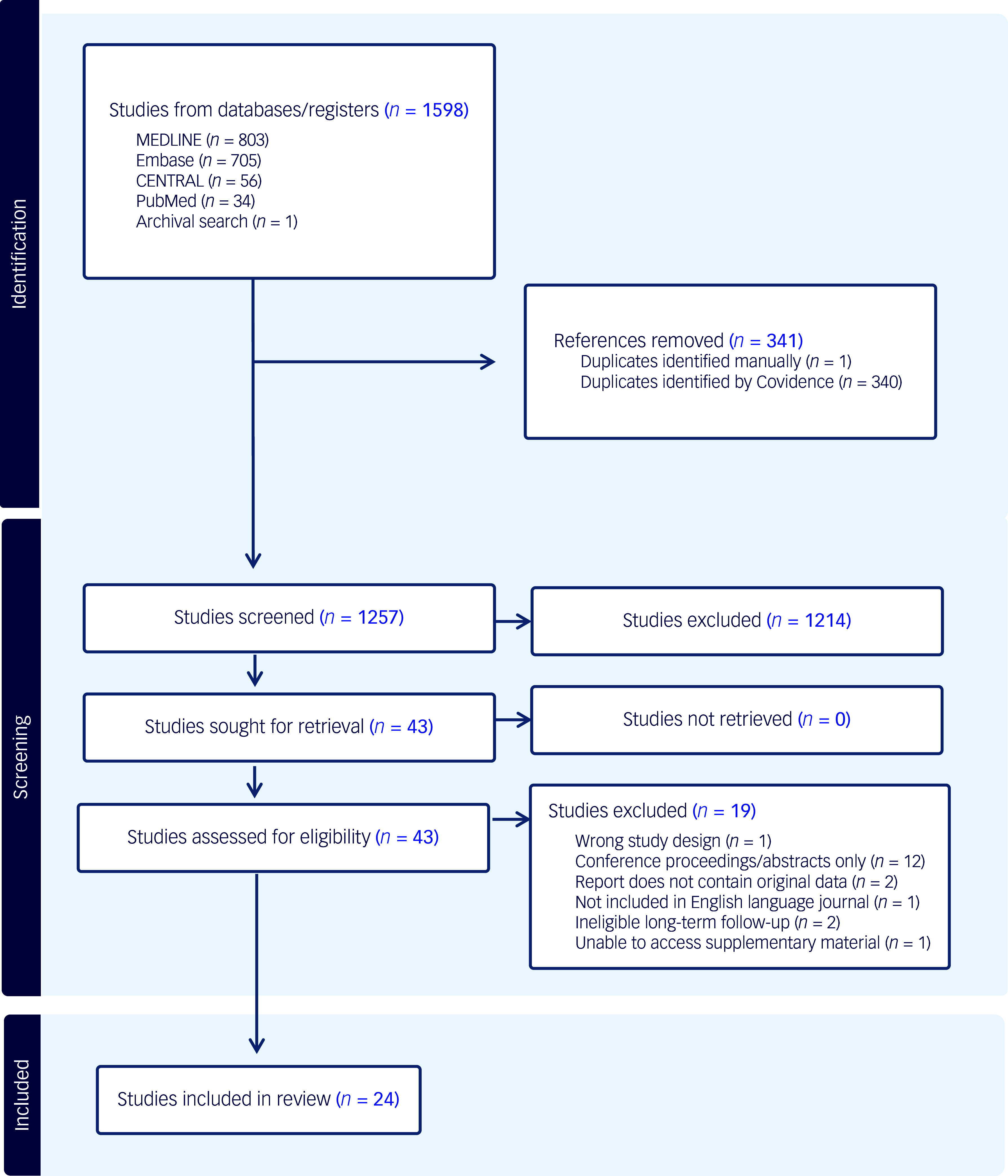
PRISMA flow diagram.

A total of 1598 studies were identified and imported for initial screening. Although 25 were eligible, the supplementary material of one paper containing adverse events data could not be retrieved despite our best efforts to contact the authors.^34^ Twenty-four studies were therefore included in this review following full-text screening, with a total of 917 unique participants (Fig. 1).^4–12,35–51^ The characteristics of eligible studies are presented in Table 2 (a fuller version of the results can be found in Supplementary Table 3).

**Table 2:** Summary of key characteristics

Study	Sample characteristics	Study design
Aaronson et al;^51^ NCT05029466	Bipolar type II with a depressive episode longer than 3 months, *N =* (9F, 6M), mean age 37.8 years (s.d. 11.6), range 18–65	Single arm, open label; phase 2; psilocybin 25 mg + psychotherapy
Aaronson et al;^49^ NCT04433858	MDD, treatment-resistant, *N =* 12 (6F, 6M), mean age 40.6 years (s.d. 9.6), range not specified	Single arm, open label; phase 2; psilocybin 25 mg + psychotherapy
Agrawal et al;^36^ NCT04593563	People with cancer with MDD, *N =* 30 (9M, 21F), mean age 56.1 years (s.d. 12.4), range 30–78	Single arm, open label; phase 2; psilocybin 25 mg + group therapy
Anderson et al;^37^ NCT02950467	Self-identified HIV-positive gay men, moderate–severe demoralization, *N =* 18 (18M), mean age 59.2 years (s.d. 4.4), range 50–66	Single-arm, open-label pilot; phase 2; psilocybin 0.3-0.36 mg/kg + group therapy
Bogenshutz et al;^4^NCT01534494	Alcohol dependence, DSM-IV, *N =* 10 (4F, 6M), mean age 40.1 years (s.d. 10.3), range 25–56	Open-label pilot; phase 2; psilocybin (0.3 mg/kg session 1, 0.3–0.4 mg/kg session 2) + psychotherapy
Back et al;^48^ NCT05163496	Clinicians with symptoms of depression, burnout, and PTSD, *N =* 30 (15F, 15M), mean age 38 years, s.d. not specified, range 29–60	RCT; double-blind; phase 2; psilocybin 25 mg + psychotherapy *v.* niacin 100 mg
Bogenshutz et al;^38^ NCT02061293	Alcohol dependence, DSM-IV, *N =* 95 (42F, 53M), mean age 45.8 years (s.d. 11.6), range not specified	Parallel RCT; double-blind; phase 2; psilocybin (25 mg/70 kg session 1, 25, 30 or 40 mg/70 kg session 2) + psychotherapy *v.* diphenhydramine (50 mg session 1, 100 mg session 2)
Carhart-Harris et al^6^	MDD, treatment-resistant, *N =* 12 (6F, 6M), mean age 42.7 years (s.d. 10.2), range 30–64	Open-label pilot; phase 2; psilocybin (10 mg session 1, 25 mg session 2) + psychological support
Carhart-Harris et al^5^	MDD, treatment-resistant, *N =* 20 (6F, 14M), mean age 44.1 years (s.d. 11.0), range 27–64	6-month follow-up of Carhart-Harris et al^6^
Carhart-Harris et al;^39^ NCT03429075	MDD, *N =* 59 (20F, 39M), mean age 41.2 years (s.d. 10.9), range 21–64	Parallel RCT; double-blind; phase 2; psilocybin group (two 25 mg psilocybin sessions + 6 weeks daily placebo) + psychological support *v.* escitalopram group (two 1 mg psilocybin sessions + 6 weeks daily oral escitalopram 10–20 mg) + psychological support
Davis et al;^40^NCT03181529	MDD, *N =* 27 randomized, 24 completed and analysed (16F, 8M), mean age 39.8 years (s.d. 12.2), range not specified	Parallel RCT; blinded clinician raters for primary outcome; phase 2; immediate treatment group – psilocybin (20 mg/70 kg session 1; 30 mg/70 kg session 2) + psychological support *v.* delayed treatment group – 8-week delay followed by psilocybin (20 mg/70 kg session 1; 30 mg/70 kg session 2) + psychological support
Ellis et al;^50^ NCT04433858	MDD, treatment-resistant veterans, *N =* 14 (2F, 13M), mean age 43.2 years (s.d. 10.9), range not specified	Open-label pilot; phase 2; psilocybin 25 mg + psychotherapy
Gukasyan et al^43^	MDD, *N =* 27 randomised, 24 completed 2 psilocybin doses and 12 month follow up (16F, 8M), mean age 39.8 years (s.d. 12.2), range not specified	12-month follow-up of Davis et al^41^
Griffiths et al;^41^ NCT00465595	Cancer patients with anxiety/depression, *N =* 56 randomized, 51 analysed, mean age 56.3 years (s.e.m. 1.4), range not specified	Cross-over double-blind; phase 2; high-dose condition 22 or 30 mg/70 kg + psychological support *v.* low (placebo-like) dose 3 mg/70 kg + psychological support
Goodwin et al;^11^ NCT03775200	MDD, treatment-resistant, *N =* 233 (121F, 112M), mean age 39.8 years (s.d. 12.2), range not specified	Parallel RCT; double-blind; phase 2; single-dose psilocybin (10 mg or 25 mg) + psychological support *v.* single-dose psilocybin (1 mg) + psychological support
Grob et al;^42^ NCT00302744	Cancer patients (advanced stage) with anxiety, *N* = 12 (11F, 1M), average age not specified, range 36–58	Crossover RCT; double-blind phase 2; single-dose psilocybin 0.2 mg/kg + psychological support *v.* niacin 250 mg + psychological support
Johnson et al^7^	Cigarette smokers, *N* = 15 (5F 10M), mean age 51.0 years (s.d. 10.5), range 26–65	Open-label pilot; phase 2; psilocybin (20 mg/70 kg session 1 and 20–30 mg/70 kg sessions 2 and 3) + smoking cessation CBT
Moreno et al^8^	OCD, *N =* 9 (2F, 7M), mean age 40.9 years (s.d. 13.2), range 26–62	Quasi-experimental; phase 2; single escalating psilocybin dose sessions: LD (100 µg/kg), MD (200 µg/kg) and HD (300 µg/kg) + psychological support with single randomized VLD session (25 µg/kg) inserted in double-blind fashion after the first dose
Peck et al;^9^ NCT04661514	Anorexia nervosa, *N =* 10 (10F), mean age 28.3 years (s.d. 3.7), range not specified	Open label; phase 2; single-dose psilocybin 25 mg + psychological support
Raison et al;^12^ NCT03866174	MDD, *N =* 104 (52F, 52M), mean age 41.1 years (s.d. 11.3), range not specified	RCT; double-blind and blinded clinician raters for primary outcome; phase 2; single-dose psilocybin 25 mg + psychological support *v.* niacin 100 mg + psychological support
Ross et al;^44^ NCT00957359	Cancer patients with anxiety/depression, *N* = 31 randomized, *n* = 29 analysed (18F, 11M), mean age 56.3 years (s.d. 12.9), range 22–75	Crossover RCT; double-blind; phase 2; psilocybin condition 0.3 mg/kg + psychotherapy *v.* niacin condition 250 mg + psychotherapy
Schneier et al;^10^ NCT04656301	SSRI resistant body dysmorphic disorder, *N* = 12 (8F, 4M), mean age 34.3 years (s.d. 8.9), range not specified	Open-label pilot; phase 2; single-dose psilocybin 25 mg + psychotherapy
Sloshower et al;^45^ NCT03554174	MDD, *N* = 22 enrolled, *n* = 19 analysed (13F, 6M), mean age 42.8 (s.d. 13.8), range 20–61	Fixed-order placebo-controlled trial; double-blind; phase 2; psilocybin condition (0.3 mg/kg) + psychotherapy *v.* inactive placebo + psychotherapy
von Rotz et al;^46^ NCT03715127	MDD, *N =* 52 (33F, 19M), mean age 36.8 (s.d. total not specified), range not specified	Parallel RCT; double-blind phase 2; single-dose psilocybin group (0.215 mg/kg) + psychotherapy (*n* = 26) *v.* placebo group (mannitol) + psychotherapy (*n* = 26)

Study characteristics including information on measurement of adverse events presented in full in Supplementary Table 3.

*N* or *n*, number of participants; F, female; M, male; s.d., standard deviation; MDD, major depressive disorder; DSM-IV, Diagnostic and Statistical Manual of Mental Disorders (4th Ed); PTSD, post-traumatic stress disorder; RCT, randomized controlled trial; s.e.m., standard error of the mean; CBT, cognitive behaviour therapy; OCD, obsessive–compulsive disorder; LD, low dose; MD, medium dose; HD, high dose; VLD, very low dose; SSRI, selective serotonin reuptake inhibitor.

### Adherence to CONSORT recommendations

**Fig. 2:**
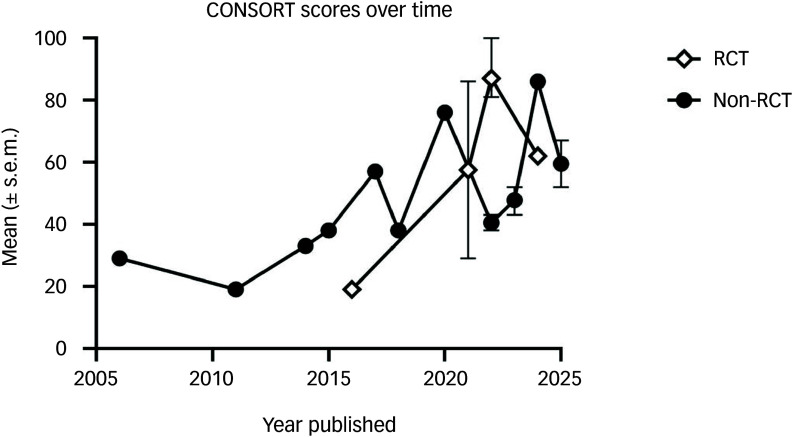
Consolidated Standards of Reporting Trials (CONSORT) quality of harm reporting scores over time. s.e.m., standard error of the mean; RCT, randomised controlled trial.

Of the 24 trials, 9 were RCTs and 15 were non-randomised, of which 2 were long-term follow-up studies. Ratings against the CONSORT Harm reporting recommendations are presented in Tables 1 and 3. Seven studies (5 RCTs, 2 non-RCTs) showed adequate adherence to the CONSORT Harms 2004 recommendations (>70%; Table 1), with 1 meeting all 21 criteria.^12^ Of the 9 RCTs, 3 were rated as very low quality (33%), 1 as moderate (11%) and 5 as high quality (56%). Of the 15 non-RCTs, 2 were rated as very low quality (13%), 9 were low quality (60%), 3 were moderate quality (20%) and 1 was high quality (7%). The THRS across all studies was 11 (i.e. low quality), with a minimum of 4 and a maximum of 21 (Table 3). Reporting quality appeared to improve over time (Fig. 2). Median adherence across all trials was 50% (range 19–100%). Agreement between raters was near perfect (Cohen’s kappa 0.95).

**Table 3:** Total harm reporting score result for CONSORT for harms items

Randomised controlled trials															
CONSORT item	Ross et al^44^		Griffiths et al^41^		Davis et al^40^	Back et al^48^	Bogenschutz et al^38^		von Rotz et al^46^		Goodwin et al^11^		Carhart-Harris et al^39^		Raison et al^12^
1	0		0		0	0	1		1		1		1		1
2	0		0		0	0	0		0		0		0		1
3a	0		0		0	1	1		1		1		1		1
3b	0		1		1	1	1		1		1		1		1
3c	0		0		0	1	1		0		1		1		1
4a	0		0		1	1	1		1		1		1		1
4b	1		0		0	1	1		1		1		1		1
4c	0		0		0	1	1		1		1		1		1
4d	0		0		0	1	1		1		1		1		1
5a	0		0		0	0	1		1		1		1		1
5b	0		0		0	0	0		0		1		0		1
6a	1		1		0	1	0		1		1		1		1
6b	1		0		1	1	1		1		1		1		1
7a	0		0		1	1	1		1		1		1		1
7b	0		0		0	1	1		1		1		1		1
8a	0		1		0	0	1		1		1		1		1
8b	0		0		0	1	1		0		1		1		1
8c	0		0		0	0	0		1		0		1		1
9	1		1		1	1	1		1		1		1		1
10a	0		0		0	0	1		1		1		1		1
10b	0		0		1	0	1		1		0		0		1
THRS	4		4		6	13	17		17		18		18		21
Ad%	19		19		29	62	81		81		86		86		100
Quality	Vlow		Vlow		Vlow	Mod	High		High		High		High		High
Non-randomised trials															
CONSORT item	Grob et al^42^	Moreno et al^8^	Johnson et al^7^	Carhart-Harris et al^5a^	Bogenschutz et al^4^	Gukasyan et al^43a^	Sloshower et al^45^	Peck et al^9^	Agrawal et al^36^	Schneier et al^10^	Aaronson et al^49^	Carhart-Harris et al^6^	Ellis et al^50^	Anderson et al^37^	Aaronson et al^51^
1	1	1	0	0	1	1	0	1	1	1	0	1	1	1	0
2	0	0	0	0	0	1	0	0	0	0	0	0	0	0	1
3a	0	0	0	1	0	0	0	0	1	0	0	1	0	0	1
3b	1	1	1	1	1	1	1	1	1	1	1	1	1	1	1
3c	0	0	0	0	0	1	0	0	0	0	0	0	1	1	1
4a	1	1	0	0	1	1	0	0	0	1	1	0	1	1	1
4b	0	0	1	1	1	1	1	1	1	1	1	0	1	1	1
4c	0	0	0	1	0	0	0	0	0	0	0	1	0	1	1
4d	0	0	0	1	0	0	0	0	0	0	0	1	0	0	1
5a	0	0	0	0	0	0	1	1	1	0	0	0	0	1	1
5b	0	0	0	0	0	0	0	0	0	0	0	0	0	0	1
6a	1	0	0	0	0	0	1	1	1	1	1	1	1	1	1
6b	0	0	0	0	0	1	1	1	0	1	1	1	1	1	1
7a	0	0	0	0	0	0	0	1	0	0	1	0	1	1	0
7b	0	1	1	1	1	0	1	1	1	1	0	1	1	1	1
8a	0	1	1	1	1	0	1	1	1	1	1	1	1	1	1
8b	0	0	1	0	1	1	1	0	1	1	1	1	1	1	1
8c	0	0	0	0	0	0	0	0	0	0	1	1	1	1	0
9	0	1	1	1	1	1	1	1	1	1	1	1	1	1	1
10a	0	0	1	0	0	0	0	0	0	0	1	0	1	1	1
10b	0	0	0	0	0	0	0	0	0	1	0	0	0	0	1
THRS	4	6	7	8	8	9	9	10	10	11	11	12	14	16	18
Ad%	19	29	33	38	38	43	43	48	48	52	52	57	67	76	86
Quality	Vlow	Vlow	Low	Low	Low	Low	Low	Low	Low	Low	Low	Mod	Mod	Mod	High

CONSORT, Consolidated Standards of Reporting Trials; THRS, total harm reporting score; Ad%, adherence %; Vlow, very low; Mod, moderate.

a. Long-term follow-up study.

### Risk of bias

With side-effects specified as the outcome of interest, risk of bias was analysed with the RoB 2 tool for the 9 RCTs, all of which were rated as having a high risk of bias (Supplementary Fig. 1). This was largely due to domain 4, ‘measurement of the outcome’, which assesses whether ‘knowledge of the assigned intervention could influence participant-reported outcomes’.^32^ Given that, across studies, participants self-reported adverse events to researchers and no study demonstrated successful blinding – a notoriously difficult problem for studies of medications such as psilocybin with obvious subjective effects – this item was rated as having a high risk of bias across studies. Agreement between raters was high (Cohen’s kappa 0.84).

### Adverse events reported in publications versus on ClinicalTrial.gov

Ten of the 24 studies could be audited for adverse events reported in publications compared with those in the corresponding CTR. Of 14 studies that could not be audited, 5 had no CTR and 9 had a CTR but no results had been uploaded as of 3 February 2025.

Of the ten studies that were audited, seven did not report the total number of adverse events or the total number of participants experiencing an adverse event. The audit revealed small inconsistencies between adverse events reported in publications and CTR (Supplementary Table 2). There was, however, no apparent systematic underreporting of side-effects in the published reports versus CTR, as previously identified for other psychiatric interventions.^26^ Inconsistencies identified were variable, including the numbers and types of adverse events reported and descriptions of specific adverse events (Supplementary Table 2) for further information. Variability in approaches to side-effects reporting made direct comparison for most studies challenging.

## Discussion

We characterised the quality of side-effects reporting in clinical trials of PAP for psychiatric disorders. We found that the body of evidence on PAP was variable in terms of quality of side-effects reporting, with 14 of 24 reports rated as being of either low or very low quality against the CONSORT harms guidelines, 4 as moderate and 6 as high quality. With side-effects as the outcome of interest, all RCTs had a high risk of bias. We also found several small inconsistencies between adverse events recorded in published reports and those in CTR, without evidence of systematic underreporting of adverse events in this body of literature.

These findings indicate somewhat superior side-effects reporting in the PAP literature compared with similar bodies of evidence. A recent evidence synthesis by our group found that none of 13 MDMA-AP trials met the criteria for adequate adherence to the CONSORT Harms recommendations, with a median adherence rate of 50%.^25^ Similar median rates were observed in the literature on esketamine (48%)^26^ and SSRIs (50%).^27^ We report a similar median adherence (50%, range 19–100) in the PAP literature; however, side-effects reporting in 6 of 24 PAP studies (i.e. 25%) was of high quality, 42% of studies met criteria for adequate adherence (70%) and 1 met all 21 criteria.^12^ Notably, all six studies with high-quality side-effects reporting were published since 2020, with some explicitly seeking to address limitations in previous trials, including in side-effects reporting.^11,12^ One possible explanation for the apparent increase in quality of side-effects reporting (Fig. 2) over time could be the publication of updated reporting standards by CONSORT in 2023.^30^ Additionally, early studies have been subject to critiques regarding methodological failings and associated issues with interpretability of findings,^52^ potentially leading to researchers designing more rigorous trials that include comprehensive reporting of adverse events. These are promising signs for strengthening of side-effects reporting in this field, and suggest that publications identified as high quality in relation to side-effects reporting should be viewed as a benchmark for future research. Evidence from these reports – particularly those RCTs that provide a higher level of evidence – should also be preferentially employed in policy decisions, and by clinicians seeking to inform patients about the potential risks of PAP.

Risk of bias was assessed using the RoB 2 tool for the 9 RCTs. Despite psilocybin-assisted psychotherapy being increasingly recognised as a potential treatment for several psychiatric indications, the limited number of RCTs published to date highlights the urgent need for more high-quality research to clarify its safety and efficacy. All nine RCTs were found to have a high risk of bias, which is similar to bias ratings in a recent MDMA-AP systematic review,^25^ where seven of eight trials were rated as high risk, with one having ‘some concerns’. Consistent with these findings, all esketamine trials included in a similar review^26^ were rated as having ‘some concerns’. Across these bodies of research, domain 4, ‘measurement of the outcome’, was the most likely to be violated, with functional unblinding presenting an ongoing issue in studies involving medications such as psychedelics that have strong psychoactive and subjective drug effects.^53^ This issue is compounded by the much-noted media hype surrounding these interventions.^54–56^ Attempts to ameliorate functional unblinding will require systematic methodological modifications in future clinical trials,^52,53^ with the aim of improving the certainty of evidence about PAP and related modalities.

Our final analysis examined adverse events reported in CTR compared with those reported in the corresponding published articles. Overall, our review revealed no evidence of systematic underreporting as suggested in a review of trials of esketamine.^26^ However, direct comparison between CTR and publications was challenging due to variability in the approaches to side-effects reporting across CTR and corresponding publications, with variations in the metrics used to quantify adverse events (i.e. total number of adverse events versus number of participants experiencing individual adverse events), timelines and thresholds for adverse events reporting. This lack of consistency in reporting itself highlights the need for more standardised approaches.

One issue that this review is unable to address is whether current approaches to identifying side-effects adequately capture some of the unique characteristics of psychedelic drugs such as psilocybin, and the potential impacts of their combination with psychotherapy. In particular, the potential for interpersonal harms occurring in PAP has been noted,^57–59^ given the increased vulnerability of patients undergoing psychotherapy while affected by psychoactive drugs. How best to ensure that these rare but profoundly harmful events are prevented and, when they do occur, documented has yet to be determined. Moreover, the content of the psychotherapy delivered was often poorly defined in the studies reviewed. Improved transparency in the description of therapeutic models would enable future reviews to better assess how specific treatment components may influence the type, frequency and severity of harms. Similarly, there is little consensus as to how phenomena such as existential or spiritual crises related to psychedelic exposure^60^ are best understood within the biomedical framework, communicated to patients and managed in PAP research and clinical practice. Of additional concern, suicidality has been noted as an adverse event in some trials;^37,41^ this may partly reflect the research focus on major depression. However, there is some indication of dose dependence in suicidality,^11^ suggesting a causal effect of the treatment itself. Considering this, careful baseline screening, close monitoring throughout the trial and robust post-treatment support are crucial to ensure patient safety. Future studies should further investigate suicidality, and other similar safety issues, by examining each event systematically, tracking adverse events and how any changes in behaviour might be linked to the therapeutic process or drug effects. More broadly, efforts to stratify adverse events reporting by participant risk group or clinical indication are limited by the current evidence base. While such stratification could reveal important differences in reporting quality or adverse events profiles, particularly across populations with differing levels of baseline vulnerability, this remains difficult due to the small number of studies per indication and inconsistent reporting standards. A better understanding of these safety issues is critical for guiding future research, implementation decisions and clinical practice.

The findings of this review highlight variability and some limitations in side-effects reporting within PAP clinical trials. While most studies included adverse events information and some (25%) were rated as high quality regarding adherence to the CONSORT Harms guidelines, the overall quality of reporting was inadequate (median adherence 50%). Similar shortcomings have been observed in related research,^25,27^ suggesting that side-effects reporting in psychiatry more broadly needs to be improved. Of note, however, is the apparent improvement in side-effects reporting in PAP trials published since 2020. To continue to enhance the quality of side-effects reporting, we echo our previous recommendation that a stronger emphasis be made on following the CONSORT Harms recommendations during peer review.^25^ Regarding PAP specifically, although recent trials show improvements in reporting, there remain some limitations in the overall body of evidence. From the clinical perspective, physicians seeking to provide information to patients considering PAP about its risk:benefit ratio should prioritise information from RCTs identified as having high-quality side-effects reporting. Moreover, patients should be informed of the relative uncertainty of existing evidence on potential side-effects given the risk of bias in existing studies. In future research, consistent and rigorous reporting practices, alongside methodological enhancements to reduce risk of bias, are essential to guide the safe translation of psilocybin-assisted psychotherapy into clinical practice.

## Supporting information

Marinis et al. supplementary material 1 Marinis et al. supplementary material

Marinis et al. supplementary material 2 Marinis et al. supplementary material

## Data Availability

All available data are contained within the submitted work.

## Supplementary material

The supplementary material is available online at https://doi.org/10.1192/bjo.2025.10847
