# Supplementary material for: Reporting of side-effects in clinical trials of psilocybin-assisted psychotherapy for psychiatric conditions: systematic review
Source: BJPsych Open. 2025 Nov 3;11(6):e261. doi: 10.1192/bjo.2025.10847 (PMC12641409; doi:10.1192/bjo.2025.10847)
Supplement: Marinis et al. supplementary material 1 — Marinis et al. supplementary material [file S2056472425108478sup001.docx]

**SUPPLEMENTAL MATERIAL:**

Data extraction:

All data were manually extracted from published papers and their corresponding ClinicalTrial.gov entry. The following information was extracted from published reports: 1) authors; 2) year published; 3) study title; 4) design; 5) control conditions; 6) blinding procedure; 7) number of participants randomised; 8) primary outcome measure; 9) inclusion and exclusion criteria; and 10) ClinicalTrial.gov entry link. Data extracted from both ClinicalTrial.gov Registers and publications included (where possible): 1) total number of participants; 2) participants with at least one adverse event; 3) total number of serious adverse events; 4) frequency threshold for reporting other adverse events; 5) total number of other (not serious) adverse events.

**Table S1: Search strategy**

| **Embase:** | 1 exp randomized controlled trial/  2 controlled clinical trial/  3 random$.ti,ab.  4 randomization/  5 intermethod comparison/  6 placebo.ti,ab.  7 (compare OR compared OR comparison).ti,ab.  8 ((evaluated OR evaluate OR evaluating OR assessed OR assess) AND (compare OR compared OR comparing OR comparison)).ab.  9 (open adj label).ti,ab.  10 ((double OR single OR doubly OR singly) adj (blind OR blinded OR blindly)).ti,ab.  11 double blind procedure/  12    parallel group$1.ti,ab.  13 (crossover OR cross over).ti,ab.  14 ((assign$ OR match OR matched OR allocation) adj5 (alternate OR group$1 OR intervention$1 OR patient$1 OR subject$1 OR participant$1)).ti,ab.  15 (assigned OR allocated).ti,ab.  16 (controlled adj7 (study OR design OR trial)).ti,ab.  17 (volunteer OR volunteers).ti,ab.  18 human experiment/  19 trial.ti.  20 or/1-19  21 (random$ adj sampl$ adj7 ("cross section$"  OR questionnaire$1 OR survey$ OR database$1)).ti,ab. NOT (comparative study/ OR controlled study/ OR randomi?ed controlled.ti,ab. OR randomly assigned.ti,ab.)  22   cross‐sectional study/ NOT (exp randomized controlled trial/ OR controlled clinical trial/ OR controlled study/ OR randomi?ed controlled.ti,ab. OR control group$1.ti,ab.)  23 (((case adj control$) AND random$) NOT randomi?ed controlled).ti,ab.  24 systematic review.ti,ab. NOT (trial.ti. OR study.ti)  25 (nonrandom$ NOT random$).ti,ab.  26 "random field$".ti,ab.  27 (random cluster adj3 sampl$).ti,ab.  28 (review.ab. AND review.pt.) NOT trial.ti.  29 ("we searched".ab. AND (review.ti. OR review.pt.))  30 "update review".ab.  31 (databases adj4 searched).ab.  32 (rat OR rats OR mouse OR mice OR swine OR porcine OR murine OR sheep OR lambs OR pigs OR piglets OR rabbit OR rabbits OR cat OR cats OR dog OR dogs OR cattle OR bovine OR monkey OR monkeys OR trout OR marmoset$1).ti. AND animal experiment/  33    animal experiment/ NOT (human experiment/ OR human/)  34    or/21-33  35    20 NOT 34  36  psilocybin.mp.  37  psilocibin.mp.  38  psilocybine.mp.  39  36 OR 37 OR 38  40   35 AND 39 |
| --- | --- |

| **Medline:** | 1 psilocybin.mp. 1753  2 psilocibin.mp. 1  3 psilocybine.mp. 97  4 1 or 2 or 3 1758  5 randomized controlled trial.pt 601043  6 controlled clinical trial.pt. 95430  7 randomized.ab. 619517  8 placebo.ab. 241997  9 drug therapy.fs. 2627495  10 randomly.ab. 417964  11 trial.ab. 667053  12 groups.ab. 2578005  13 5 or 6 or 7 or 8 or 9 or 10 or 11 or 12 5769813  14 4 and 13 571  15 exp animals/ not humans.sh. 5160002  16 14 not 15 557 |
| --- | --- |

**
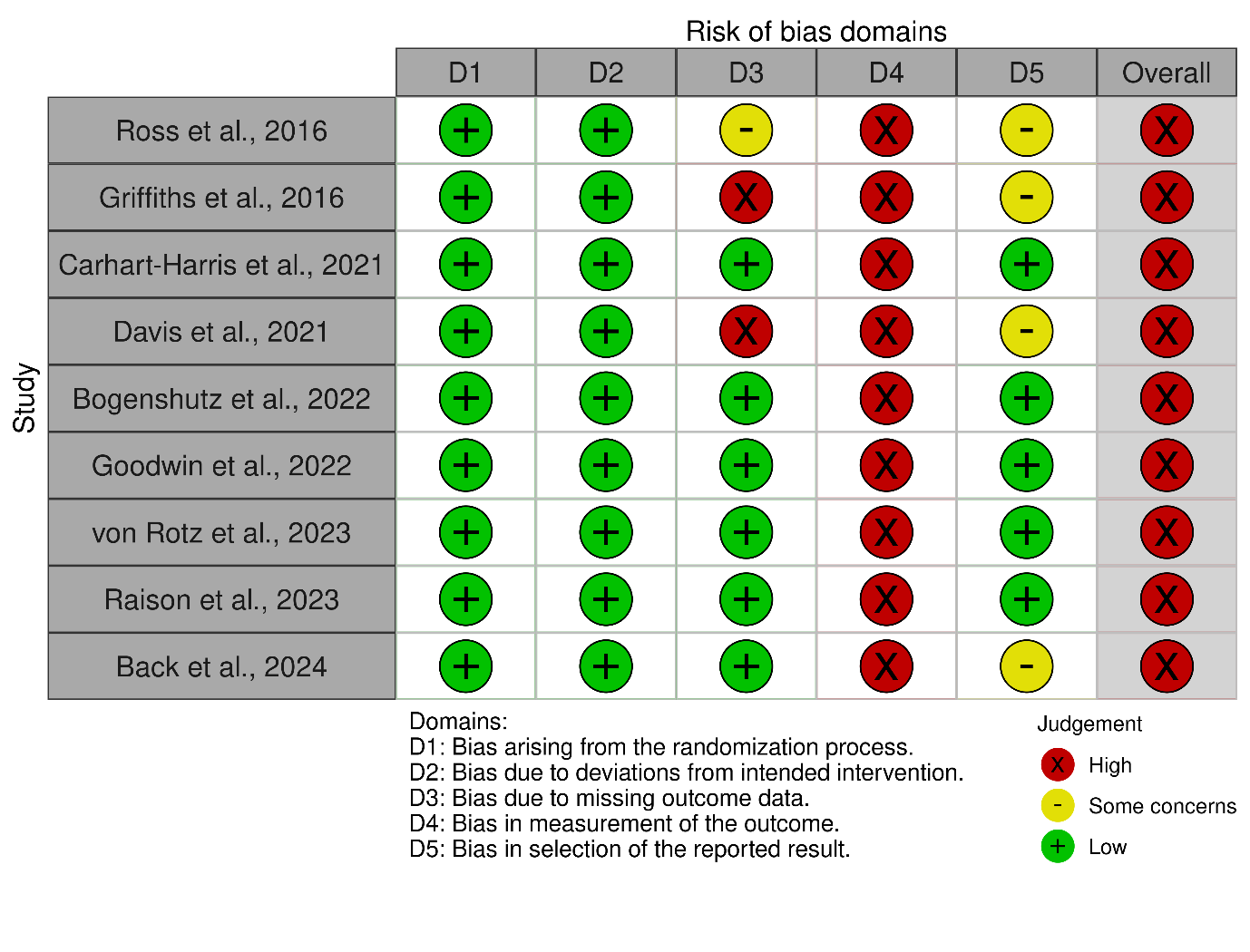
Supplementary Figure 1. Risk of Bias Assessment for Randomized Controlled Trials using the RoB-2 tool**

**Table S2: Published Article and CTR Comparison**

| **Study** | **Differences** |
| --- | --- |
| **Anderson et al., (2020)** | SAEs: no notable differences between PA and CTR  Other AEs:   - PA does not report total number of AEs or participants experiencing AEs, so cannot directly compare with total numbers in CTR - No differences identified in the description of AEs and number of participants who experienced each AE type |
| **Bogenshutz et al., (2015)** | SAEs: nil reported  Other AEs:   - PA only reports TRAEs - PA does not report total number of AEs or participants experiencing AEs, so cannot directly compare with total numbers in CTR - No differences identified in the number of participants reported for each AE type - CTR reported 6 AE types (10 events) that were not reported in PA   - Additional AEs: haematuria, depression, alcohol withdrawal syndrome, upper respiratory tract infection, forearm fracture, and road traffic accident   - Likely unreported in PA as deemed not to be treatment related |
| **Bogenshutz et al., (2022)** | SAEs:   - No differences in number of participants who experienced SAEs - Description of one SAE differed between PA and CTR:   - PA reported n = 1 (in diphenhydramine control group) had 2 psychiatric admissions due to suicidal ideation during binge drinking episodes   - CTR described same event as ‘n = 1 suicide attempt’   Other AEs:   - PA reported total number of adverse events, CTR reported total number of people who had any AE, so could not be directly compared - No differences identified in the number of participants reported for each AE type |
| **Carhart Harris et al. (2021)** | SAEs: Nil reported  Other AEs:   - No differences in total number of participants experiencing AEs - PA reports more types of AE than those reported in CTR   - PA additionally reported over 50 types of AEs   - PA reported a subset of AEs (those reported in 3 or more participants) and all AEs separately   - CTR only reported the subset of AEs, but reported a frequency threshold of 0% |
| **Davis et al., (2021)** | SAEs: Nil reported  Other AEs:   - Difference identified in timeframe of AE data collection   - PA - Within two weeks after each dosing session (4 weeks total)   - CTR - From first dosing session to one week following second dosing session (3 weeks total) - PA does not report total number of AEs or participants experiencing AEs, so cannot directly compare with total numbers in CTR - No differences identified in the number of participants reported for each AE type - PA reported 1 AE type that was not reported in CTR   - CTR and PA both reported number of headaches after session, PA separately reported headaches during session (reported in n = 16) |
| **Griffiths et al., (2016)** | SAEs:   - PA reported mortality in n = 4 before 6-month follow-up (n = 3 due to progression of their cancer, n = 1 suicide). All-cause mortality was omitted from the CTR so could not be compared - PA reported no SAEs related to psilocybin administration. CTR reported no SAEs (i.e., did not report mortality data)   Other AEs:   - PA does not report total number of AEs or participants experiencing AEs, so cannot directly compare with total numbers in CTR   - PA only reported the percentage of participants who experienced each type of AE - Differences between percentages of participants who experienced each AE type reported in PA and CTR   - PA reported higher rates of elevated systolic blood pressure, nausea or vomiting, and anxiety than CTR   - Discrepancy may have been due to using different denominators for the percentage calculation. The CTR reports all randomised (n = 56). The paper does not specify the adverse event n, however other outcome measures were only reported in those who completed both dosing sessions (n = 50) - PA reports more types of AE than those reported in CTR   - PA also additionally reported physical discomfort, headaches, psychological discomfort, and paranoid   - May be in part explained by that the CTR frequency threshold for reporting of 5%, however some of these events occurred in over 5% of the sample per the paper |
| **Goodwin et al., (2022)** | SAEs:   - PA reported total numbers of combined SAEs and AEs across whole study, and total SAEs separately for each time point of the study, so cannot be compared to total SAEs across entire study reported in CTR - One SAE reported in CTR but not in PA (adjustment disorder with anxiety)   Other AEs:   - PA reported total numbers of combined SAEs and AEs across whole study, and number of participants reporting each AE that occurred in ≥5% of participants in any group, split by three timepoints (day 1, day 2-week 3k, week 3-week). The CTR reports total of each AE (both n and event) with a reporting threshold of 5% across whole trial. Since one is split by time point, and the other across the entire study, the n participants who experienced each type of AE can also not be compared. |
| **Peck et al., (2023)** ^a^ | SAEs: Nil reported  Other AEs:   - PA does not report total number of AEs or participants experiencing AEs, so cannot directly compare with total numbers in CTR - Differences identified in description of type of AE data reported   - PA reported on number of participants who had each TRAE and safety measures at day 1 (post-treatment)   - CTR reported number of participants that had any AE up until 14 weeks - CTR reports more types of AE than those reported in PA   - CTR additionally reports diarrhoea, concussion, fall, blood pressure orthostatic and radial nerve compression - PA reported a participant experiencing suicidal ideation at the 3 month follow up, which was deemed not related to study follow-up (not reported in CTR) |
| **Ross et al., (2016)** | SAEs: Nil reported  Other AEs:   - PA does not report total number of AEs or participants experiencing AEs, so cannot directly compare with total numbers in CTR   - PA only reported the percentage of participants who experienced the most common medical AEs related to psilocybin - PA reports more types of AE and higher rates of AEs than those reported in CTR   - CTR only reports 2 types of AEs and only in the psilocybin group (elevations in BP and HR, in 3.45% of the sample, transient anxiety in 3.45% of sample)   - PA reported elevated BP in 76% of the sample and transient anxiety in 28% of the sample   - PA also reported headaches/migraines, nausea, and transient psychotic-like symptoms as common AEs attributed to psilocybin, these were not reported in the CTR |
| **Schneier et al., (2023)** | SAEs: Nil reported  Other AEs: Nil differences |

***AE****: Adverse Event,* ***BP:*** *Blood Pressure,* ***CTR:*** *Clinical Trial Register,* ***HR:*** *Heart Rate,* ***PA:*** *Published Article,* ***SAE:*** *Serious Adverse Event,* ***TRAE:*** *Treatment-Related Adverse Event*

^a^*CTR quality control review process not concluded*
