# Supplementary material for: Reporting of side-effects in clinical trials of psilocybin-assisted psychotherapy for psychiatric conditions: systematic review
Source: BJPsych Open. 2025 Nov 3;11(6):e261. doi: 10.1192/bjo.2025.10847 (PMC12641409; doi:10.1192/bjo.2025.10847)
Supplement: Marinis et al. supplementary material 2 — Marinis et al. supplementary material [file S2056472425108478sup002.docx]

**Table S3: Summary of Key Characteristics, Adverse Event Reporting, and Outcomes Across Studies**

| **Study** | **Sample Characteristics** | **Psilocybin Condition** | **Control Condition** | **Blinding Procedures** | **Study Phases** | **AE Measurement** | **AEs Recorded in Publication** (n = number of participants experiencing AE) |
| --- | --- | --- | --- | --- | --- | --- | --- |
| **Aaronson et al (2024);** single arm, open-label; phase 2; NCT05029466 | Bipolar Type II with a depressive episode longer than 3 months, N = 15 (9F, 6M), Mean age = 37.8 (SD = 11.6), Range = 18–65 | Psilocybin 25 mg + psychotherapy | N/A | N/A | Screening and preparation, psilocybin session, 12-week psychotherapy with 1 dosing session, 12-week follow-up | AEs assessed by asking about side effects at each time point, AEs coded using MedDRA, causality and severity assignment by PI according to pre-specified definitions | No SAEs. Only reported the most common AE: headache (n=4) |
| **Aaronson et al (2025);** single arm, open-label; phase 2; NCT04433858 | MDD, treatment-resistant, N = 12 (6F, 6M), Mean age = 40.6 (SD = 9.6), Range = not specified | Psilocybin 25 mg + psychotherapy | NA | NA | Screening and preparation, psilocybin session, 12-week psychotherapy with 1 dosing session, 12-week follow-up | AEs assessed by direct observation by lead therapist, in addition to clinical interviews by the study physician | No SAEs. AEs: Headache (n=2), insomnia (n=2), psychomotor agitation (n=1), worsening depression symptoms (n=1) |
| **Agrawal et al (2023);** single arm, open-label; phase 2; NCT04593563 | People with cancer with MDD, N = 30 (9M, 21F), Mean age = 56.1 (SD = 12.4), Range = 30–78 | Psilocybin 25 mg + group therapy | N/A | N/A | Screening and preparation, psilocybin session, group integration, 8-week follow-up | Observed and spontaneously reported AEs recorded at each time point. Investigator assessed intensity, causality, seriousness | No treatment-related SAEs. Common AEs^a^ : Headache (n=24), nausea (n=12), Crying (n=8), altered mood (n=8) |
| **Anderson et al (2020);** single-arm, open-label pilot; phase 2; NCT02950467 | Self-identified HIV-positive gay men, moderate-severe demoralization, N = 18 (18M), Mean age = 59.2 (SD = 4.4), Range = 50–66 | Psilocybin 0.3-0.36 mg/kg + group therapy | N/A | N/A | Screening and preparation, 2 weeks group therapy, 1 psilocybin session, 2-3 weeks group therapy, 3-month follow-up | Observed and spontaneously reported AE recorded at each time point; vital signs assessed at regular intervals; assessed AE presence and severity in psilocybin sessions with ChEQ; AE’s classified by the NIH DAIDS Table for Grading the Severity of Adult and Paediatric Adverse Events v2.0. | SAEs (n=3): Cholecystitis, renal cell carcinoma, stimulant-induced psychosis, suicide attempt, pneumothorax (unrelated). Common AEs^a^: Hypertension (n=12), anxiety (n=8), headache post-session (n=8). n=14 expected adverse reactions, 7 of which were considered severe. n=2 unexpected, related reactions, 1 PTSD flashback, 1 anxiety with methamphetamine relapse |
| **Bogenshutz et al (2015);**  Open label pilot; phase 2; NCT01534494 | Alcohol dependence, DSM-IV, N = 10 (4F, 6M), Mean age = 40.1 (SD 10.3), Range = 25-56 | Psilocybin, 0.3 mg/kg (session 1) and psilocybin, 0.3-0.4 mg/kg (session 2) + psychotherapy | N/A | N/A | Screening and preparation, 12-week psychotherapy with 2 dosing sessions, 24-week follow-up | AE case report forms at sessions and follow-ups; vital signs at each visit and medication session | Only reported TRAEs. No SAEs. AEs: Nausea/emesis (n=1), IBS and diarrhea (n=1), mild headache (n=5), insomnia (n=1). |
| **Back et al (2024);** RCT; phase 2;  **NCT05163496** | Clinicians with symptoms of depression, burnout, and PTSD, N = 30 (15F, 15M), Mean age = 38, SD = not specified, Range = 29-60 | Psilocybin 25 mg + psychotherapy | Niacin 100 mg | Double-blind; integrity assessed in participants at end of dosing session (100% correct) | Screening and preparation, 4-week psychotherapy with 1 dosing session, 6-month follow-up | AEs assessed by asking about side effects during medication session and each integration visit; spontaneous and observed AEs also documented; causality and severity assignment according to pre-specified definitions; included information from other safety measures (C-SSRS, vitals) | No SAEs. AEs: mild nausea (n=4), mild headache (n=4), mild tachycardia (n=2), mild hypertension (n=6), moderate hypertension (n=8), severe hypertension (n=1), transient suicidal thoughts (n=1) |
| **Bogenshutz et al (2022);** parallel RCT; phase 2; NCT02061293 | Alcohol dependence, DSM-IV, N = 95 (42F, 53M), Mean age = 45.8 (SD = 11.6), Range: Not specified | Psilocybin 25 mg/70 kg (session 1), 25, 30 or40 mg/70 kg (session 2) + psychotherapy | Diphenhydramine, 50mg (session 1), 100 mg (session 2) | Double-blind; integrity assessed in participants (1^st^ session 93.6% correct; 2^nd^ session 94.7% correct) and therapists (1^st^ session 92.4% correct; 2^nd^ session 97.4% correct) | Screening and preparation, 12-week psychotherapy with 2 dosing sessions, 24-week follow-up | AE case forms; vital signs at each visit and medication session | 204 AEs reported: 119 in psilocybin, 85 in diphenhydramine. SAEs: 3 in diphenhydramine group in n=2 (2 events of psychiatric admission due to suicidal ideation in n=1, 1 Mallory-Weiss tear in n=1). AEs that were more frequently occurring in psilocybin group compared to diphenhydramine (p < 0.05): Headache (n=21), nausea (n=10), anxiety (n=7) |
| **Carhart-Harris et al (2016);** open-label pilot; phase 2 | MDD, treatment-resistant, N = 12 (6F, 6M), Mean age = 42.7 (SD = 10.2), Range = 30–64 | Psilocybin 10 mg (session 1), and 25 mg (session 2) + psychological support | N/A | N/A | Screening and preparation, 2 dosing sessions (7 days apart), 3-month follow-up | Method and timing of AE assessment unclear; causality and severity assignment by PI according to pre-specified definitions | No SAEs. 30 AEs reported across n = 12. Common AEs^a^ included transient anxiety (n=12), confusion/thought disorder (n=9), headache (n=4) |
| **Carhart-Harris et al (2018);** 6-month follow-up from **Carhart-Harris et al (2016);** open-label pilot; phase 2 | MDD, treatment-resistant, N = 20 (6F, 14M), Mean age = 44.1 (SD = 11.0), Range = 27–64 | Psilocybin 10 mg (session 1), and 25 mg (session 2) + psychological support | N/A | N/A | Screening and preparation, 2 dosing sessions (7 days apart), 3-month follow-up; additional 3-month follow-up | AEs assessed by asking about side effects at each post-treatment visit; spontaneous and observed AEs also documented; causality and severity assignment by PI according to pre-specified definitions | No SAEs. Did not report total number of AEs. Common AEs^a^: transient anxiety (n=15), headaches (n=8), nausea (n=5), paranoia (n=3) |
| **Carhart-Harris et al (2021);** parallel RCT; phase 2; NCT03429075 | MDD, N = 59 (20F, 39M), Mean age = 41.2 (SD: 10.9 not specified), Range = 21–64 | Psilocybin group (n=30); two 25mg psilocybin sessions + 6 weeks of daily placebo + psychological support | Escitalopram group (n=29; two 1mg psilocybin sessions + 6 weeks of daily oral escitalopram (10-20mg) + psychological support | Double-blind | Screening and preparation, 2 dosing days (3 weeks apart) alongside 6 weeks daily placebo or escitalopram | AEs assessed by open-ended question and observation at every visit; coded using MedDRA; causality and severity assignment by PI according to pre-specified definitions | No SAEs reported; AEs reported by n=50 (n=26 in psilocybin group, n=24 in escitalopram group); common AEs^a^ in psilocybin group included headache (n=20), nausea (n=8), migraine (n=3); common AEs^a^ in escitalopram group included anxiety (n=4) and dry mouth (n=4) |
| **Davis et al (2021);**  parallel RCT; phase 2; NCT03181529 | MDD, N = 27 randomized, 24 completed and analysed (16F, 8M), Mean age = 39.8 (SD = 12.2), Range: Not specified | Immediate treatment group (n=15 randomized, n=13 analysed). Psilocybin 20mg/70kg (session 1), and 30mg/70kg (session 2) + psychological support | Delayed treatment group (n=12 randomized, n=11 analysed). 8-week delay followed by psilocybin 20mg/70kg (session 1), and 30mg/70kg (session 2) + psychological support | Blinded clinician raters for primary outcome only | Screening and preparation, 8-week intervention with two dosing days (1-3 weeks apart). Either immediately or after an 8-week wait list delay | Unclear how AEs were assessed during sessions. Participants were asked about occurrence of headache on the day after each session. Participant asked if they had experienced any other AEs at follow-up visits. Unclear how AEs were attributed to the study drug | No SAEs reported; Only reported challenging emotional/physical “adverse effects” during session from the ChEQ, cardiovascular and headache events during session, and AES reported within 2 weeks of each session that were rated by staff as possibly related to psilocybin. Most common AE reported during session was headache (16/48 sessions) |
| **Ellis et al (2025);** open-label pilot; phase 2; NCT04433858 | MDD, treatment-resistant veterans, N = 14 (2F, 13M), Mean age = 43.2 (SD = 10.9), Range = not specified | Psilocybin 25 mg + psychotherapy | N/A | N/A | Screening and preparation, psilocybin session, 12-week psychotherapy with 1 dosing session, 12-week follow-up | AEs assessed virtually by study psychiatrist at all study visits | No SAEs. AEs: mild-moderate headache (n=7), nausea (n=3), retroperistalsis (n=2), mild back pain (n=2), increased psychological distress (n=2), worsening tinnitus (n=1), suicidal ideation during antidepressant taper (n=1) |
| **Gukasyan et al (2022);** 12-month follow-up from **Davis et al. (2021;)** RCT; phase 2 | MDD, N = 27 randomised, 24 completed 2 psilocybin doses and 12 month follow up (16F, 8M), Mean age = 39.8 (SD = 12.2), Range: Not specified | Immediate treatment group (n=15 randomized, n=13 analysed and completed long term follow-up). Psilocybin 20mg/70kg (session 1), and 30mg/70kg (session 2) + psychological support | Delayed treatment group (n=12 randomized, n=11 analysed and completed long-term follow-up). 8-week delay followed by psilocybin 20mg/70kg (session 1), and 30mg/70kg (session 2) + psychological support. | Blinded clinician raters for primary outcome only | Screening and preparation, 8-week intervention with 2 dosing days (1-3 weeks apart). Either immediately or after an 8-week waiting list delay + 12-month follow-up | AEs recorded at each follow-up timepoint. Unclear how AEs were assessed or attributed to the study drug | No SAEs reported. 1 AE reported potentially related to study drug (one-minute-long episode of visual distortions about 3 months following second psilocybin session) |
| **Griffiths et al 2016;** cross-over double-blind; phase 2; NCT00465595 | Cancer patients with anxiety/ depression, N = 56 randomized, 51 analysed, Mean age = 56.3 (SEM = 1.4), Range: Not specified | High dose condition 22 or 30mg/70kg + psychological support | Low (placebo-like) dose condition 1 or 3mg/70kg + psychological support | Double-blind; high/low dose integrity assessed in study monitors (88% correct) | Screening and preparation, high/low dose psilocybin sessions (counterbalanced cross-over), approximately 5 weeks between sessions, 6-month follow-up | Unclear how AEs were assessed or attributed to the study drug | No SAEs attributed to psilocybin reported. 4 deaths reported (3 cancer progression, 1 suicide 11 days post-low-dose psilocybin session); common AEs ^a^: BP elevation (n=17 of total participants elevated systolic BP, n=7 elevated diastolic BP in high dose), psychological discomfort n=16 in high dose), and physical discomfort (n=11 in high dose) |
| **Goodwin et al (2022);** parallel RCT; phase 2; NCT03775200 | MDD, treatment resistant, N = 233 (121F, 112M), Mean age = 39.8 (SD=12.2), Range: Not specified | Single dose 10 mg psilocybin (n=79) or 25 mg psilocybin (n=75) + psychological support | Single dose 1mg psilocybin (n=79) + psychological support | Double-blind | Screening and preparation, single psilocybin session, 12 week follow up | AE case form tracked description, timing, severity, and relation to IP; included information from other safety measures such as C-SSRS and vitals. Assessed each visit, coded using MedDRA, causality and severity assignment by PI according to pre-specified definitions | SAEs reported in n=8 between day 2 – week 3 (25mg n=4, 10mg n=4), including suicidal ideation (25mg n=2, 10mg n=2), intentional self-injury (25mg n=2, 10mg n=1 ), and hospitalization (10mg n=1). SAEs reported in n=8 between week 3 – week 12 (25mg n=4, 10mg n=3, 1mg n=1), including suicidal behaviour (25mg n=3), ideation (10mg n=1), intentional self-injury (10mg n=1, 1mg n =1), adjustment disorder (25mg n=1), depression (10mg n=1), and drug withdrawal (25mg n=1). Any AE reported in n=179 (25mg n=66, 10mg n=56, 1mg n=57). Common AEs ^a^: in 25mg group were headache, nausea, dizziness, fatigue. |
| **Grob et al (2011);** crossover RCT; phase 2; NCT00302744 | Cancer patients (advanced stage) with anxiety, N=12 (11F, 1M). Average age: not specified, Range = 36-58 | Psilocybin condition; Single dose 0.2 mg/kg psilocybin + psychological support | Placebo condition; Niacin 250 mg + psychological support | Double-blind. Participants were aware of the two conditions, but not the group initially assigned. | Screening and preparation, 2 medication sessions (randomized cross-over) several weeks apart, 6-month follow-up | Unclear how AEs were assessed or attributed to the study drug | AE data unclear; minor HR and BP elevations reported after psilocybin administration (unclear number of participants affected), no adverse psychological effects reported |
| **Johnson et al (2014);** open-label pilot; phase 2 | Cigarette smokers, N=15 (5F 10M). Mean age = 51.0 (SD = 10.5), Range = 26-65 | Psilocybin 20mg/70kg (session 1) and high doses 20-30mg/70kg (session 2 and 3) + smoking cessation CBT | N/A | N/A | Screening and preparation, 15-week smoking cessation treatment with 3 psilocybin sessions (session 1 and 2 two weeks apart, optional session 3 six weeks after), 6-month follow-up | Safety data derived from cardiovascular outcomes, SOCQ, headache ratings, and Visual Effects Questionnaire. Unclear if recorded spontaneously reported AEs | No "clinically significant AEs requiring intervention" reported. SOCQ n=6/15 strong-extreme fear, fear of insanity, or feeling trapped. BP and HR elevation without specifying number of affected participants. For the 10 participants assessed n=8 reported at least one post-session headache |
| **Moreno et al (2006);** Quasi-experimental; phase 2 | OCD; N = 9 (2F, 7M); Mean age = 40.9 (SD = 13.2), Range = 26 - 62 | Single escalating psilocybin dose sessions: LD (100 µg/kg), MD (200 µg/kg), and HD (300 µg/kg) + psychological support | Single VLD session (25 µg/kg). | Dose-escalation with randomized VLD inserted in double-blind fashion after the first dose (LD). | Screening and preparation, 4 psilocybin sessions separated by at least 1-week, 24-hour follow-up | Unclear how AEs were assessed or attributed to the study drug | Transient HR and BP elevations reported after psilocybin administration n=1. n=2 experienced discomfort with being hospitalized leading to study withdrawal. No other AEs were reported |
| **Peck et al (2023);** open-label; phase 2; NCT04661514 | Anorexia nervosa, N = 10 (10F); Mean age = 28.3 (SD = 3.7), Range: Not specified | Single dose psilocybin 25mg + psychological support | N/A | N/A | Screening and preparation,  single dosing visit followed by 84-day follow-up period | Unclear how AEs were assessed or attributed to the treatment; AEs were recorded from baseline to one week after dosing | No SAEs reported. Only TEAEs were reported. The most common ^a^ TEAE reported were headache (n=8), fatigue (n=7) and nausea (n=3) |
| **Raison et al (2023);** RCT; phase 2; NCT03866174 | MDD, N = 104 (52F, 52M); mean age = 41.1 (SD = 11.3), Range: Not specified | Single dose psilocybin 25mg + psychological support (n=51) | 100mg niacin + psychological support (n=53) | Double blind; Participants and study site personnel blinded to treatment group. Blinded central raters used to assess primary outcome (MADRS). | Screening and preparation. Single dosing session. 43-day follow-up | AE case form tracked description, timing, severity, and relation to IP; spontaneously reported/observed AEs recorded as well as solicited AEs measured by specific assessments (e.g. visual perceptual effects, suicidal ideation, heart rate, and blood pressure). Assessed each visit, coded using MedDRA, causality and severity assignment by PI according to pre-specified definitions | No treatment related SAEs reported. 3 SAEs reported between enrolment and randomization (nephrolithiasis, obstructive hernia, and appendicitis). In the psilocybin group, n=44 had at least one AE, compared to n=33 in the niacin group. TRAEs were higher in the psilocybin group (n=41) versus in niacin (n=24). Common AEs^a^ in the psilocybin group included headache (n=33), nausea (n=24), and visual perceptual effects on dosing day (n=22) |
| **Ross et al (2016);** crossover RCT; phase 2; NCT00957359 | Cancer patients with anxiety/ depression, N=31 randomized, n=29 analysed, (18F, 11M), Mean age = 56.3 (SD = 12.9), Range = 22-75 years | Psilocybin condition 0.3mg/kg + psychotherapy | Niacin condition 250mg + psychotherapy | Double-blind. Blinding integrity was assessed in therapists 28/29 (97%) correct. | Screening and preparation.  2 medication sessions (randomized cross-over) one week apart, 26-week follow-up | Unclear how AEs were assessed or attributed to the study drug; AEs monitored throughout the trial, and cardiovascular measures assessed during medication sessions. Only AEs and SAEs attributed to medication were reported | No SAEs reported. Only most common AEs from psilocybin group were reported, including n=22 BP/HR increase, n=8 headaches/migraines, and n=5 transient anxiety |
| **Schneier et al (2023);** open-label pilot; phase 2; NCT04656301 | SSRI resistant body dysmorphic disorder, N=12 (8F, 4M), Mean age = 34.3 (SD = 8.9), Range: Not specified | Single dose psilocybin 25mg + psychotherapy | N/A | N/A | Screening and preparation. Single psilocybin session, 12-week follow-up | Open-ended query at each visit; transient non-serious experiences during dosing were only counted if lasting over 8 hours. | No SAEs reported. Mild AEs reported in n=11 including fatigue (n=5), headache (n=3), and nausea (n=2) |
| **Slowshower et al (2023);** fixed-order placebo-controlled trial; phase 2; NCT03554174 | MDD, N=22 enrolled, n=19 analysed, (13F, 6M), Mean age 42.8 (SD = 13.8), Range = 20-61 | Psilocybin condition (0.3 mg/kg) + psychotherapy | Inactive placebo + psychotherapy | Double-blind. Participants and staff were told two of three doses would be provided: (either placebo, low, or moderate) in random order. However, placebo was always given first, followed by 0.3mg/kg psilocybin. Participants guessed condition correctly 78.9% for placebo and 80% for psilocybin | Screening and preparation. Psilocybin administered 4 weeks after placebo. n=19 received placebo and n=15 psilocybin, 12-week follow-up period | Unclear how AEs were assessed or attributed to the study drug; AEs were monitored throughout the trial, with vital signs assessed during medication sessions. | 1 SAE involving psychiatric hospitalization approximately 2 weeks following psilocybin session due of lack of improvement of depression. Total of 25 AEs across the psilocybin session and 3 AEs across placebo sessions. Common AEs^a^ in psilocybin sessions were headaches (7 AEs), anxiety (3 AEs), and dysphoria (3 AEs) |
| **von Rotz et al (2023);** parallel RCT; phase 2; NCT03715127 | MDD, N = 52 (33F, 19M), Mean age = 36.8 (SD: Total not specified), Range: Not specified | Single dose psilocybin group (0.215 mg/kg) + psychotherapy (n=26) | Placebo group (mannitol) + psychotherapy (n=26) | Double blind | Screening and preparation. Single psilocybin session, 24-week follow-up | Neutral questioning about well-being used to ascertain AEs; AE case form to record volunteered AEs and PI assessed abnormal outcomes on measures of psychological and physical health, suicidality, vital signs, weight, and medication use. Measured from pre-treatment to 14 days post-treatment: no long-term follow-up.  Causality and severity assignment made by PI according to pre-specified definitions | No SAEs reported. 11 AEs were reported, 8 in psilocybin group and 3 in placebo. AEs in the psilocybin group were headache (4 AEs), dizziness (2 AEs), nausea (1 AE), and diarrhea (1AE) |

***AE:*** *Adverse Event****, BP:*** *Blood Pressure,* ***C-SSRS:*** *Columbia-suicide Severity Rating Scale,* ***ChEQ:*** *Challenging Experiences Questionnaire*, ***DAIDS:*** *Division of AIDS,* ***DSM-IV:*** *Diagnostic and Statistical Manual of Mental Disorders, 4th Edition,* ***F:*** *Female,* ***HR:*** *Heart Rate,* ***IP:*** *Investigational Product,* ***IBS:*** *Irritable Bowel Syndrome,* ***MADRS:*** *Montgomery-Åsberg Depression Rating Scale,* ***M:*** *Male,* ***MDD:*** *Major Depressive Disorder,* ***N/n =*** *number of participants;* ***OCD:*** *Obsessive Compulsive Disorder,* ***VLD:*** *Very Low Dose,* ***LD:*** *Low Dose,* ***MD:*** *Medium Dose,* ***HD:*** *High Dose,* ***MedDRA:*** *Medical Dictionary for Regulatory Activities,* ***NIH:*** *National Institutes of Health,* ***PI:*** *Principal Investigator,* ***RCT:*** *Randomized Controlled Trial,* ***SOCQ:*** *States of Consciousness Questionnaire,* ***SAE:*** *Serious Adverse Event,* ***SD:*** *Standard Deviation,* ***SEM:*** *Standard Error of the Mean,* ***SSRI****: Selective Serotonin Reuptake Inhibitor,* ***TEAE:*** *Treatment-emergent Adverse Events,* ***TRAE:*** *Treatment-related Adverse Event*

^a^***Common AEs:*** *Are AEs most frequently reported in the publication*
